# Relationship and impact of hand segmentation in IADLs for predicting Alzheimer

**DOI:** 10.1002/alz.085259

**Published:** 2025-01-09

**Authors:** Eyitomilayo Yemisi Babatope, Mireya Saraí García‐Vázquez, Alejandro Álvaro Ramírez‐Acosta

**Affiliations:** ^1^ Instituto Politécnico Nacional, CITEDI, Tijuana, BJ Mexico; ^2^ MIRAL R&D&I Multimedia, San Diego, CA USA

## Abstract

**Background:**

A decline in Instrumental Activities of Daily Living (IADLs) indicates cognitive impairment, a marker of early detection of Alzheimer’s disease (AD). Obtaining hand information within the assessment of IADLs may be an innovative approach to predicting cognitive decline. Hands play a vital role while performing IADL and can be used in assessing human visuomotor skills. This is a non‐invasive approach to early detection and intervention. Hand segmentation techniques can help obtain hand information necessary for cognitive function assessment. This research evaluates the visuomotor skills in humans by segmenting hands in egocentric videos to obtain hand information as a feature to predict cognitive decline.

**Method:**

In this work, visual information with motor actions was measured based on hand coordination and movement. Analysis of hand information obtained from hand interaction is measured to predict Alzheimer's disease. A convolutional neural network was used to build an improved segmentation model (RefineNet‐Pix) for hand segmentation. This model was trained with a public dataset that captures hand interaction and movements using an egocentric camera (egoHands). To validate its accuracy, we tested the model on egohands test data, GTEA (Georgia Tech Egocentric Activities) datasets, and random images from the internet. We made use of less expensive kernels and more optimization techniques in performing the segmentation task as compared to existing models.

**Result:**

The result obtained from the semantic segmentation task yielded a better performance in terms of speed and accuracy when compared with the baseline model.

**Conclusion:**

Details of hand movements can point to precise changes in cognitive decline. An improved performance of the hand segmentation process with our RefineNet‐Pix can help in extracting hand information. This is a useful feature in identifying a decline in cognitive status. This is a promising, non‐invasive, and cost‐effective approach that can help clinicians in the early detection of Alzheimer's disease.

